# Importance of Social Determinants in Screening for Depression

**DOI:** 10.1007/s11606-021-06957-5

**Published:** 2021-08-17

**Authors:** Robert M. Califf, Celeste Wong, P. Murali Doraiswamy, David S. Hong, David P. Miller, Jessica L. Mega, for the Baseline Study Group

**Affiliations:** 1 Verily Life Sciences, San Francisco, CA USA; 2 Department of Psychiatry and Behavioral Sciences and the Duke Institute for Brain Sciences, Duke University School of Medicine, Durham, NC USA; 3 Department of Psychiatry and Behavioral Sciences, Stanford University School of Medicine, Stanford, CA USA

**Keywords:** Patient Health Questionnaire-9, measures of health and disease, effective clinical intervention

## Abstract

**Abstract:**

**Importance:**

The most common screening tool for depression is the Patient Health Questionnaire-9 (PHQ-9). Despite extensive research on the clinical and behavioral implications of the PHQ-9, data are limited on the relationship between PHQ-9 scores and social determinants of health and disease.

**Objective:**

To assess the relationship between the PHQ-9 at intake and other measurements intended to assess social determinants of health.

**Design, Setting, and Participants:**

Cross-sectional analyses of 2502 participants from the Baseline Health Study (BHS), a prospective cohort of adults selected to represent major demographic groups in the US; participants underwent deep phenotyping on demographic, socioeconomic, clinical, laboratory, functional, and imaging findings.

**Interventions:**

None.

**Main Outcomes and Measures:**

Cross-sectional measures of clinical and socioeconomic status (SES).

**Results:**

In addition to a host of clinical and biological factors, higher PHQ-9 scores were associated with female sex, younger participants, people of color, and Hispanic ethnicity. Multiple measures of low SES, including less education, being unmarried, not currently working, and lack of insurance, were also associated with higher PHQ-9 scores across the entire spectrum of PHQ-9 scores. A summative score of SES, which was the 6th most predictive factor, was associated with higher PHQ-9 score after adjusting for 150 clinical, lab testing, and symptomatic characteristics.

**Conclusions and Relevance:**

Our findings underscore that depression should be considered a comorbidity when social determinants of health are addressed, and both elements should be considered when designing appropriate interventions.

**Supplementary Information:**

The online version contains supplementary material available at 10.1007/s11606-021-06957-5.

Depression is a complex, chronic condition that is critical to public health and the well-being of many individuals, families, and their loved ones. Major depressive disorder affects 17 million adults in the United States (US) and more than 250 million people worldwide; less severe forms of depressed affect are even more common.^1^ Similarly, race, ethnicity, wealth, education, and other measures of social status and function are now recognized as major determinants of health outcomes.^2^

Bidirectional relationships have been reported between depression and many chronic illnesses^3^; however, most studies have focused on specific conditions, such as diabetes, stroke, or congestive heart failure, as opposed to a multidimensional deep phenotyping approach. Likewise, recent evidence suggests a bidirectional relationship between depression and poverty,^4^ adding to the robust literature on links between poverty and poor health. These findings highlight the need to better understand the possible moderating effects of social determinants and the degree to which social determinants and depression are intertwined. The most common screening tool for depression is the Patient Health Questionnaire-9 (PHQ-9), whose operating characteristics are well known^5^ and have been validated in a variety of contexts.^6,7^ Despite extensive research on the clinical and behavioral implications of PHQ-9,^8,9^ there is limited research on the relationship between the PHQ-9 score and social determinants of health.

The Baseline Health Study (BHS)^10^ is a prospective cohort study of an adult population selected to represent major demographic groups in the US. In BHS, deep phenotyping of numerous demographic, clinical, laboratory, functional, and imaging findings is coupled with ongoing longitudinal follow-up. The purpose of this study was to assess the relationship between PHQ-9 score and a broad array of measurements intended to assess social determinants of health.

## METHODS

### The Baseline Health Study

BHS methods have been previously described,^10^ including entry and exclusion criteria, the institutional review board and participant consent procedures, the data collection scheme, and key components of study procedures. BHS is enrolling a large number of participants, beginning with intensive measurement of the first 2502 people (the deeply phenotyped cohort) in whom a large volume of multimodal data are collected. Four clinical BHS sites in the US have begun enrollment.

BHS participants were enrolled through a virtual online registry; selection of participants for the deep phenotyping cohort included in this report was performed using an algorithm to produce a cohort representative of US adult age, race, and ethnicity. People in good health and with medical conditions were included and the sampling method was designed to over-represent people at risk of heart disease or cancer. The PHQ-9 in this report was collected at the initial study visit in person or online.

A pre-BHS pilot study, which tested clinical assessment workflows, was conducted in 200 healthy participants prior to initiation of the primary study. BHS is funded by Verily (San Francisco, CA) and is managed in collaboration with Stanford University (Stanford, CA), Duke University (Durham, NC), and the California Health and Longevity Institute (Westlake Village, CA) with enrolling sites in Durham, NC; Kannapolis, NC; Los Angeles, CA; and Palo Alto, CA. The extended studies have governance approaches specific to the needs of each study. Herein, we examine a cross-sectional analysis of the first BHS time point PHQ-9 scores.

### Statistical Methods

Distributional measures, medians and 25th and 75th percentiles for continuous variables, and counts and percentages for categorical variables were computed and summarized across each of 5 PHQ-9 severity groups^5^ (0, 1–4, 5–9, 10–14, >14), divided by convention to be consistent with prior studies. The Cochran-Armitage trend test for binomial variables^11,12^ and the Spearman rank correlation test for continuous variables^13^ or categorical variables that are ordinal in nature (e.g., education and income) were used to test for linear trend across severity group. Multiple tests were not adjusted for, given the exploratory nature of this study. Subsequent studies with pre-planned hypotheses are needed to confirm results.

Leveraging the breadth of data collected in BHS, penalized regression using the least absolute shrinkage and selection operator (LASSO) was conducted to select a model of physical, mental, phenotypic, symptom, and sociodemographic factors that may be predictive of the PHQ-9 score (logarithm of PHQ-9 + 1). Prior to modeling, each multi-valued (i.e., beyond binary) categorical predictor (e.g., race and smoking status) was converted using one-hot encoding. Five key socioeconomic variables (household income, education, employment status, marital status, and health insurance) collected via the Life Circumstances and Habits survey were defined as follows: (1) highest education completed: high school or less or otherwise (coded as 1/0); (2) household income: <25K or otherwise (coded as 1/0); (3) marital status: unmarried or otherwise (coded as 1/0); (4) employment status: not working or otherwise (coded as 1/0); and (5) health insurance: no or otherwise (coded as 1/0). These five variables were summed to create a socioeconomic status (SES) score, which was entered into the LASSO with all other variables listed in eTables 1.1–1.7 and eTable 2, excluding patient-reported outcome (PRO) scales in eTable 1.6. PROs, self-reported medical conditions, and self-reported symptoms related to mental health or depression have been excluded from the model to minimize less interpretable or informative findings.

LASSO regression techniques require an input dataset with complete data. Rather than case-wise deletion, missing data was primarily addressed using iterative regression-based imputation, in which values of the missing fields are predicted using a regression model based on available data from complete cases. First, the 5 key socioeconomic variables described above were included in the models with an additional “missing” level to indicate missing observations. All other missing data fields were grouped by data type and then rank-ordered by most to least missing data. The rank of the whole group was based on the amount of missingness of the majority (≥50%) of the fields within that group. At the first imputation step, grouped fields with a small amount of missing data (i.e., <2%) were imputed with the fields that were never missing (i.e., demographic and baseline characteristics). The grouped field(s) with a larger number of missing data (i.e., between 2 and <5%) were imputed using the newly imputed data from the first step plus the fields without missing data. The remaining imputation steps continued in an iterative process until all missing data fields were imputed.

Data were randomly split into a training set (approximately 70% of the data), which was used to build the models, and an independent test set, which was used to evaluate model performance. A 10-fold cross-validation of LASSO was carried out using only the training data to produce an optimal tuning parameter (the minimum value of lambda), and the final linear model was then trained on the full training set, retaining all predictors with coefficients not equal to zero. Since inferential statistics for LASSO are subject to bias,^14^ a linear regression with the retained predictors from LASSO was conducted to estimate inferential statistics.

A partial correlation network was developed in order to depict the complex interrelationships among PHQ-9, SES, and other health measures. A correlation matrix containing all variables listed in eTables 1.1–1.7 and eTable 2 was generated, and only the top 10–15 variables from each predictor group (e.g., medical conditions, symptoms, labs) that were most correlated with PHQ-9 (based on the correlation coefficient) were included in the network to enhance legibility.

## RESULTS

The relationship between the PHQ-9 score and key demographic characteristics is shown in Table 1. Female sex, younger participants, people of color, and those of Hispanic ethnicity had higher PHQ-9 scores. Table 2 displays socioeconomic status: less education, lower income, non-married, not currently working, and lack of insurance were all associated with higher PHQ-9 scores. Table 3 shows the relationship between PHQ-9 scores and other scales reflecting psychological and social distress. The inter-relationship of these different (but related) measures of distress is evident across the spectrum of measures. By multiple measures, lower SES was associated with higher PHQ-9 score.

**Table 1:** Demographics: PHQ-9 Score

	PHQ-9 0 (N=484)	PHQ-9 1–4 (N=1086)	PHQ-9 5–9 (N=518)	PHQ-9 10–14 (N=184)	PHQ-9 15+ (N=93)
Age, median (25th, 75th)*	53.6 (36.7, 66.1)	51.7 (37.0, 66.5)	47.6 (32.7, 60.7)	42.8 (31.2, 55.1)	42.4 (32.1, 54.2)
Female sex*	227 (46.9)	622 (57.3)	299 (57.7)	108 (58.7)	62 (66.7)
Race					
Black	80 (16.5)	153 (14.1)	82 (15.8)	31 (16.8)	19 (20.4)
White	294 (60.7)	731 (67.3)	323 (62.4)	110 (59.8)	57 (61.3)
Asian†	64 (13.2)	113 (10.4)	47 (9.1)	15 (8.2)	5 (5.4)
NHOPI	7 (1.4)	11 (1)	7 (1.4)	2 (1.1)	0 (0.0)
American Indian or Alaska Native	4 (0.8)	8 (0.7)	11 (2.1)	3 (1.6)	1 (1.1)
Other†	35 (7.2)	70 (6.4)	48 (9.3)	23 (12.5)	11 (11.8)
Ethnicity					
Hispanic	54 (11.2)	108 (9.9)	69 (13.3)	26 (14.1)	13 (14.0)
Site					
Los Angeles	94 (19.4)	194 (17.9)	111 (21.4)	35 (19)	21 (22.6)
Durham	99 (20.5)	196 (18.0)	102 (19.7)	40 (21.7)	25 (26.9)
Kannapolis	100 (20.7)	226 (20.8)	105 (20.3)	41 (22.3)	28 (30.1)
Palo Alto†	191 (39.5)	470 (43.3)	200 (38.6)	68 (37.0)	19 (20.4)

Data shown are no. (%), unless otherwise indicated

*NHOPI*, Native Hawaiians and other Pacific Islanders; *PHQ-9*, Patient Health Questionnaire-9

P values for trend were calculated with the use of Spearman correlation or Cochrane-Armitage tests, where appropriate

*P value for trend <0.0001. †P value for trend <0.01

**Table 2:** Socioeconomic Characteristics: PHQ-9

	PHQ-9 0 (N=484)	PHQ-9 1–4 (N=1086)	PHQ-9 5–9 (N=518)	PHQ-9 10–14 (N=184)	PHQ-9 15+ (N=93)
Education*					
High school or less	32.0 (6.6)	72.0 (6.6)	50.0 (9.7)	25.0 (13.6)	21.0 (22.6)
Some college	77.0 (15.9)	222.0 (20.4)	140.0 (27.0)	54.0 (29.3)	35.0 (37.6)
College	141.0 (29.1)	345.0 (31.8)	150.0 (29.0)	44.0 (23.9)	20.0 (21.5)
Post-graduate	186.0 (38.4)	361.0 (33.2)	127.0 (24.5)	42.0 (22.8)	12.0 (12.9)
Income, $*					
Under 25,000	28.0 (5.8)	63.0 (5.8)	61.0 (11.8)	33.0 (17.9)	33.0 (35.5)
25,000 to 50,000	42.0 (8.7)	125.0 (11.5)	71.0 (13.7)	37.0 (20.1)	14.0 (15.1)
50,000 to 100,000	104.0 (21.5)	247.0 (22.7)	124.0 (23.9)	39.0 (21.2)	24.0 (25.8)
100,000 to 150,000	76.0 (15.7)	174.0 (16.0)	63.0 (12.2)	17.0 (9.2)	5.0 (5.4)
150,000 to 200,000	51.0 (10.5)	113.0 (10.4)	40.0 (7.7)	16.0 (8.7)	1.0 (1.1)
Over 200,000	105.0 (21.7)	204.0 (18.8)	70.0 (13.5)	16.0 (8.7)	6.0 (6.5)
Marital status					
Married*	257.0 (53.1)	588.0 (54.1)	227.0 (43.8)	63.0 (34.2)	23.0 (24.7)
Formerly in long-term relationship	18.0 (3.7)	51.0 (4.7)	18.0 (3.5)	12.0 (6.5)	7.0 (7.5)
Living together†	41.0 (8.5)	83.0 (7.6)	58.0 (11.2)	24.0 (13.0)	13.0 (14.0)
Divorced	35.0 (7.2)	92.0 (8.5)	41.0 (7.9)	18.0 (9.8)	14.0 (15.1)
Never in long-term relationship‡	53.0 (11.0)	123.0 (11.3)	85.0 (16.4)	29.0 (15.8)	21.0 (22.6)
Separated	12.0 (2.5)	19.0 (1.7)	13.0 (2.5)	12.0 (6.5)	2.0 (2.2)
Widowed	13.0 (2.7)	37.0 (3.4)	18.0 (3.5)	6.0 (3.3)	7.0 (7.5)
Employment status					
Employed for wages	248.0 (51.2)	524.0 (48.3)	248.0 (47.9)	81.0 (44.0)	37.0 (39.8)
Self-employed	48.0 (9.9)	110.0 (10.1)	63.0 (12.2)	21.0 (11.4)	8.0 (8.6)
Not working 1 year or more*	5.0 (1.0)	18.0 (1.7)	13.0 (2.5)	7.0 (3.8)	8.0 (8.6)
Not working <1 year	7.0 (1.4)	26.0 (2.4)	17.0 (3.3)	2.0 (1.1)	5.0 (5.4)
Retired*	108.0 (22.3)	251.0 (23.1)	78.0 (15.1)	14.0 (7.6)	6.0 (6.5)
Homemaker‡	6.0 (1.2)	34.0 (3.1)	12.0 (2.3)	12.0 (6.5)	6.0 (6.5)
Student	9.0 (1.9)	25.0 (2.3)	11.0 (2.1)	13.0 (7.1)	2.0 (2.2)
Unable to work*	4.0 (0.8)	6.0 (0.6)	23.0 (4.4)	15.0 (8.2)	14.0 (15.1)
Insurance status					
Insured*	420.0 (86.8)	945.0 (87.0)	426.0 (82.2)	143.0 (77.7)	74.0 (79.6)
Not insured*	12.0 (2.5)	50.0 (4.6)	38.0 (7.3)	18.0 (9.8)	13.0 (14.0)
Smoking status					
Current*	30.0 (6.2)	118.0 (10.9)	89.0 (17.2)	40.0 (21.7)	36.0 (38.7)
Former	90.0 (18.6)	247.0 (22.7)	116.0 (22.4)	50.0 (27.2)	18.0 (19.4)
Non-smoker*	364.0 (75.2)	721.0 (66.4)	313.0 (60.4)	94.0 (51.1)	39.0 (41.9)

*PHQ-9*, Patient Health Questionnaire-9;  P values for trend were calculated with the use of Spearman correlation or Cochrane-Armitage tests, where appropriate; Data shown are no. (%)

*P value for trend < 0.0001. †P value for trend < 0.01. ‡P value for trend < 0.001

**Table 3:** Relationship Between PHQ-9 Scores and Other Scales Reflecting Psychological and Social Distress

	PHQ-9 0 (N=484)	PHQ-9 1–4 (N=1086)	PHQ-9 5–9 (N=518)	PHQ-9 10–14 (N=184)	PHQ-9 15+ (N=93)
AUDIT-C score, median (25th, 75th)	2.0 (1.0, 3.0)	2.0 (1.0, 3.0)	2.0 (1.0, 3.0)	1.0 (0.0, 3.0)	1.0 (0.0, 3.0)
PRO and ePRO, median (25th, 75th)					
Sheehan Disability Scale†	0.0 (0.0, 0.0)	0.0 (0.0, 2.0)	3.0 (0.0, 8.0)	8.0 (4.0, 13.8)	15.5 (6.2, 22.0)
GAD-7*	0.0 (0.0, 1.0)	1.0 (0.0, 3.0)	4.0 (2.0, 7.0)	8.0 (4.0, 11.0)	11.0 (6.0, 15.0)
WHODAS*	0.0 (0.0, 1.0)	1.0 (0.0, 2.0)	3.0 (1.0, 7.0)	6.0 (3.0, 12.0)	11.0 (5.0, 19.0)
BRFSS ACE*	1.0 (0.0, 2.0)	1.0 (0.0, 3.0)	2.0 (1.0, 4.0)	3.0 (1.0, 5.0)	4.0 (2.0, 6.5)
PROMIS pain intensity*	5.0 (3.0, 7.0)	6.0 (4.0, 7.0)	7.0 (5.0, 9.0)	8.0 (6.0, 10.0)	9.0 (6.0, 11.0)
PROMIS pain interference*	7.0 (6.0, 10.0)	8.0 (6.0, 12.0)	11.0 (7.0, 15.0)	14.0 (9.0, 20.0)	18.0 (12.0, 24.0)
PANAS positive affect*	38.0 (33.0, 42.0)	35.0 (31.0, 40.0)	32.0 (27.0, 36.0)	29.0 (24.0, 33.0)	27.0 (21.0, 32.0)
PANAS negative affect*	11.0 (10.0, 13.0)	13.0 (11.0, 16.0)	16.0 (12.0, 21.0)	20.5 (15.0, 25.0)	22.0 (17.0, 30.0)
Subjective happiness*	24.0 (22.0, 27.0)	23.0 (20.0, 25.0)	19.0 (16.0, 22.0)	17.0 (12.2, 20.0)	15.0 (11.0, 18.0)
Satisfaction with life*	29.0 (27.0, 32.0)	28.0 (23.0, 31.0)	23.0 (18.0, 27.0)	19.0 (13.0, 24.0)	15.0 (10.0, 20.0)
Perceived social support*	72.0 (63.0, 80.0)	71.0 (62.0, 78.0)	64.0 (55.0, 73.0)	59.0 (49.0, 71.0)	52.0 (37.8, 66.0)

*AUDIT-C*, Alcohol Use Disorders Identification Text-Concise; *BRFSS ACE*, Behavioral Risk Factor Surveillance System Adverse Childhood Experiences; *ePRO*, electronic patient-reported outcomes; *GAD-7*, General Anxiety Disorder-7; *PANAS*, positive and negative affect schedule; *PHQ-9*, Patient Health Questionnaire-9; *PRO*, patient-reported outcomes; *PROMIS*, Patient-Reported Outcomes Measurement Information System; *WHODAS*, World Health Organization Disability Assessment Schedule; Data shown are no. (%)

*P value for trend <0.0001;  †*P* value for trend <0.01

**Fig. 1:**
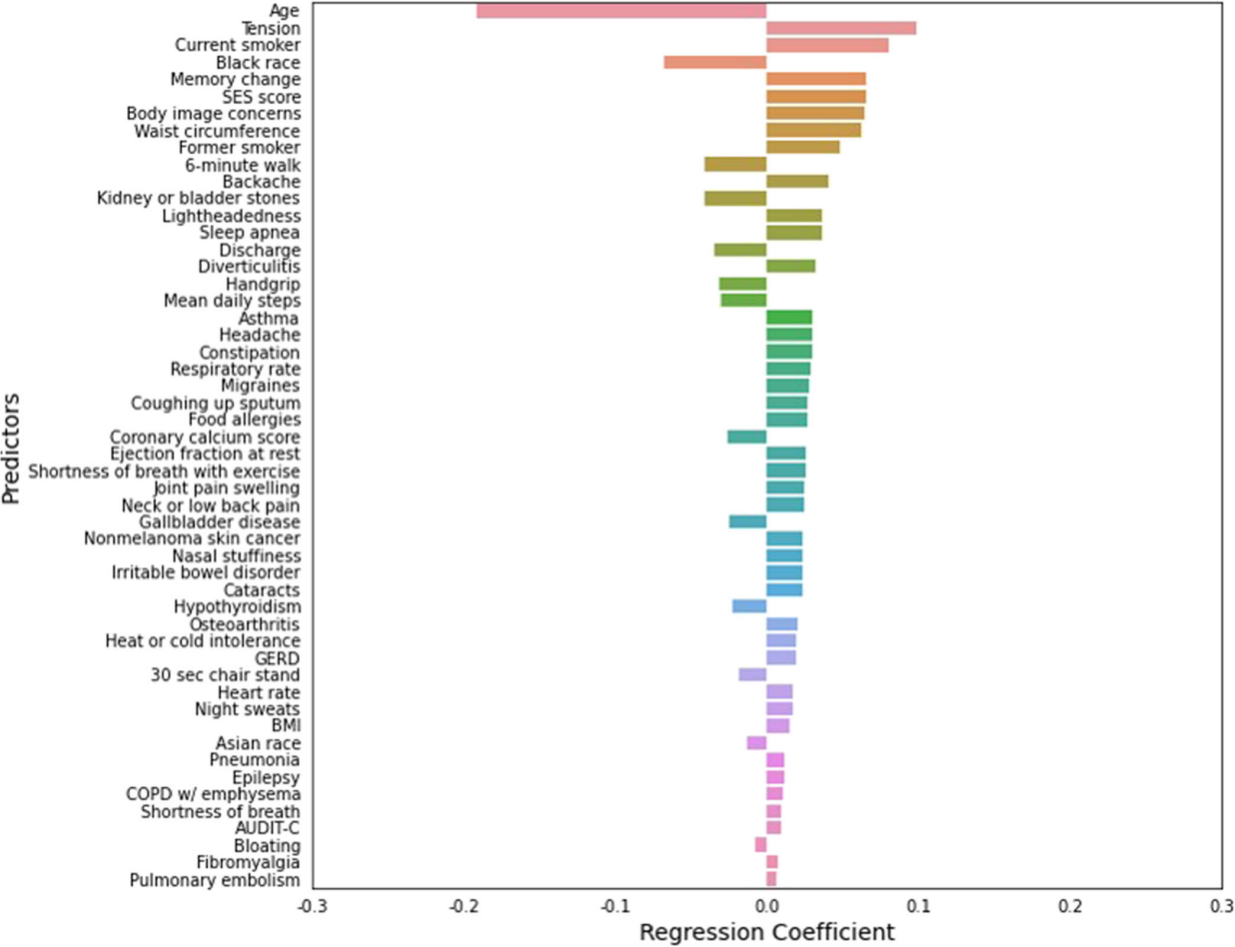
Factors associated with PHQ-9 score in linear model. SES was in the top six predictors of PHQ-9 score, significantly predicting higher PHQ-9 after adjusting for demographics, behavior, medical conditions, symptoms, and physical function (p<0.001). LASSO, least absolute shrinkage and selection operator; PHQ-9, Patient Health Questionnaire-9; SES, socioeconomic status.

In a simple unadjusted linear model, SES significantly predicted higher PHQ-9 score (p<0.001, R^2=0.08). After 10-fold cross-validation of LASSO was carried out, where model performance was similar between the training and test set, 52 variables were found to be predictive and explained 24% of the variance in PHQ-9 score. The linear model containing all retained variables from LASSO was significant (F=4.20, p<0.001), and explained 25% of the variance in PHQ-9 score. The results of the linear model are shown in Figure 1. SES was in the top six predictors of PHQ-9 score, significantly predicting higher PHQ-9 after adjusting for demographics, behavior, medical conditions, symptoms, and physical function (p<0.001). Although people of Black race had modestly higher PHQ-9 scores (Table 1), after adjusting for all other factors, Black race was associated with *lower* PHQ-9 scores (p=0.040).

**Fig. 2:**
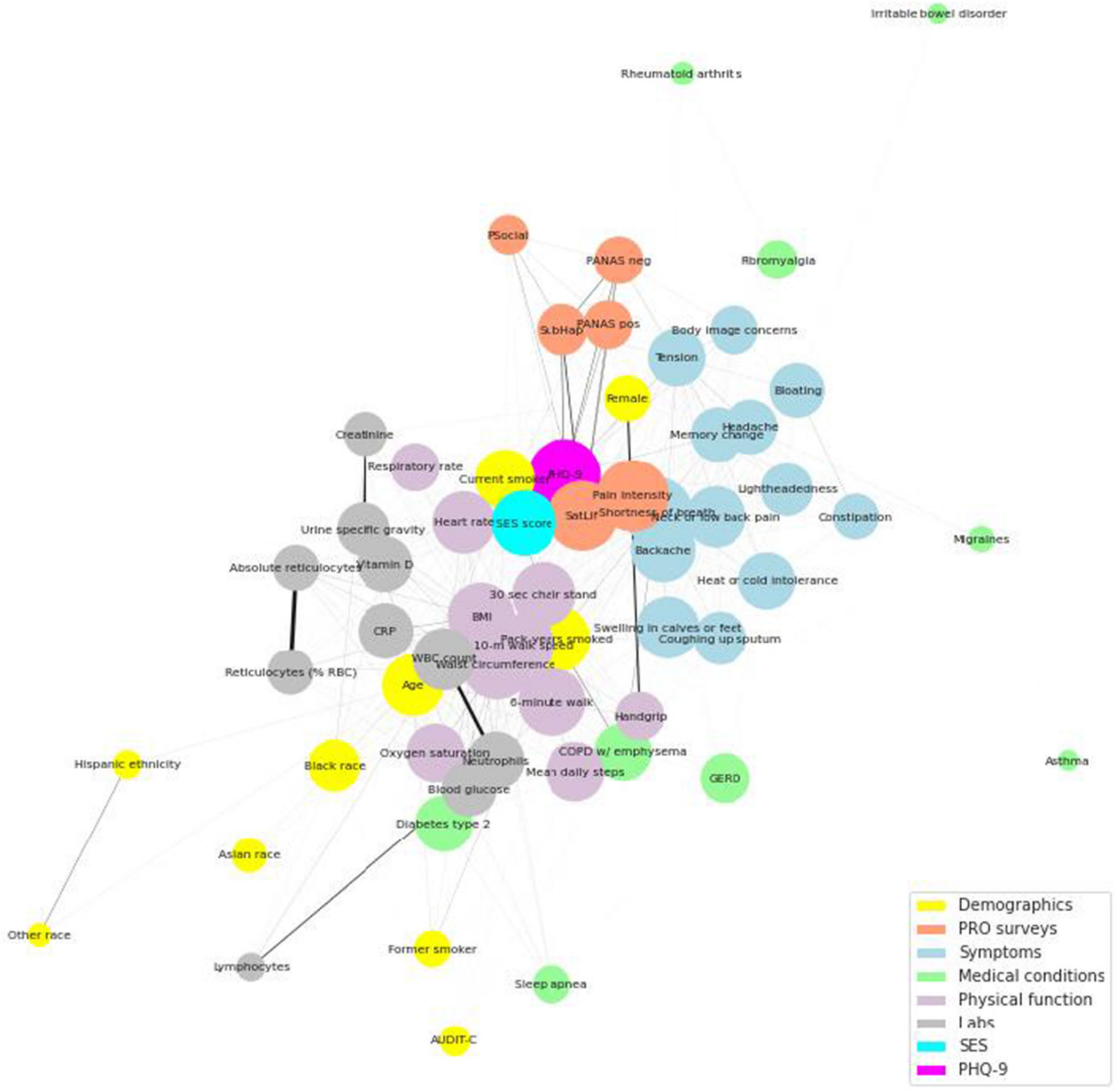
Network depicting relationship of PHQ-9 and SES with selected variables. This figure demonstrates the relationships among PHQ-9, SES, and other key measures in a partial correlation network. The length of the edges is inversely proportional to the magnitude of the correlation, and hence, highly related nodes appear closer together, with thicker edges indicating stronger correlations. PHQ-9, Patient Health Questionnaire-9; SES, socioeconomic status.

Figure 2 demonstrates the relationships among PHQ-9, SES, and other key measures in a partial correlation network. The length of the edges is inversely proportional to the magnitude of the correlation, and hence, highly related nodes appear closer together, with thicker edges indicating stronger correlations. The local clustering coefficient (a measure of a node’s connections with other nodes) of PHQ-9 is 0.52, and the eigenvector centrality (a measure of how connected a node is to other important nodes) of PHQ-9 is 0.22. The size of the nodes indicates variables with a greater number of connections with the other variables in the network. The largest connected subnetwork includes PHQ-9 and SES with laboratory variables on one side and disease symptoms and life satisfaction on the other. Race categories appear as unconnected nodes relatively distant from the center. There is also a locally connected node cluster of well-being variables (e.g., positive and negative affect schedule [PANAS], and subjective happiness) that is linked to both physical and mental health through the PHQ-9. Likewise, the locally connected subnetwork of pain and body symptoms is linked to PHQ-9 through female sex.

## DISCUSSION

Our study confirms and emphasizes previous findings in the literature regarding the relationship between PHQ-9 and SES. While these relationships are not particularly surprising, they highlight how PHQ-9 is an entree into concerns about social determinants that demand more attention. The PHQ-9 is used in clinical practice^15^ as recommended by the US Preventive Services Task Force^16^ for screening within a health system or for public health assessment, yet when performing these assessments, contextual awareness is critically important.

As a cross-sectional study, this analysis cannot answer questions of cause and effect. The ongoing BHS longitudinal study will assess PHQ-9 and detailed serial measures of biological, clinical, behavioral, and social function. This measurement depth of demographic, clinical, biological, and behavioral issues offers an opportunity to better understand how different aspects of distress track together or differ over time.

Other studies have shown the relationship between PHQ-9 score and lower income, joblessness, less education, and lack of insurance, which raises the question of how to design interventions that address all of these concerns. McClintock et al. evaluated an intervention for hypertension that included medical and social determinants and found a greater improvement in PHQ-9.^17^ Huang et al. evaluated the construct of depression in the PHQ-9 for people of different racial and ethnic backgrounds.^6^ Income was a key correlate of PHQ-9 in Nigeria.^18^ A major review of depression from the UK focused on the relevance of social determinants.^19^ Interventions solely targeting the individual, but neglecting issues of socioeconomics and social context, will likely have limited impact on depression status.^4,20–24^ Furthermore, efforts to improve social determinants should consider specific treatment for depression, since depression may limit the ability of individuals to respond to opportunity.

People of Black race had slightly higher PHQ-9 scores, but lower PHQ-9 scores after adjustment for other factors; this finding is consistent with other studies suggesting that people of Black race may have greater resilience in the face of socioeconomic factors.^25,26^ This finding was unexpected, and although it was validated in our study, it should be considered preliminary and deserves follow-up. Across the spectrum of PHQ-9 scores, the higher the score, the lower the income, educational status, or level of social connection. Since our analysis is cross-sectional, we cannot discern whether these findings represent a different approach to revealing concerns or more significant distress.

This study has some limitations. First, our examination is cross-sectional in nature. The follow-up is now accruing in the study, and the trajectory of change related to the multidimensional associations will be informative. Second, BHS participants are volunteers from selected sites who express willingness to share data, and it is likely that people with significant depression are less likely to volunteer. Finally, while the population is generally representative of adult age, sex, race, and ethnicity, it is not a fully representative sample of the population; in particular, volunteers for digital technology studies are different from volunteers found in non-digital studies.^27^ We also lack detailed information on depression treatment, a potentially modifying factor.

In conclusion, PHQ-9 scores are related to multiple measures that indicate poor SES; therefore, focusing only on depression may have limited effectiveness. Similarly, the high PHQ-9 scores associated with social and economic disparities suggest that policy and economic strategies are needed to accompany individual efforts aimed toward improving depression status. When a high PHQ-9 score or other indicators of depression bring a patient to the attention of a clinician, contextual awareness is critically important to provide an effective clinical intervention. In particular, when an individual is evaluated for depression, behavioral and social factors should be included in their holistic evaluation. Similarly, efforts to improve social and economic status of individuals and populations should consider depression as a factor in reducing the ability of individuals to respond to interventions.

## Supplementary Information

ESM 1 (DOCX 27 kb)

## References

[1] GBD 2017 Disease and Injury Incidence and Prevalence Collaborators (2018). Global, regional, and national incidence, prevalence, and years lived with disability for 354 diseases and injuries for 195 countries and territories, 1990–2017: a systematic analysis for the Global Burden of Disease Study 2017. Lancet.

[2] DeSalvo KB, Wang YC, Harris A, Auerbach J, Koo D, O’Carroll P (2017). Public health 3.0: a call to action for public health to meet the challenges of the 21st century. Prev Chroni Dis.

[3] Perry PJ. The Interaction of Major Depression and Medical Illness. Medscape; 2005. Available at: https://www.medscape.org/viewarticle/517033. Accessed 2 May 2021.

[4] Ridley M, Rao G, Schilbach F, Patel V (2020). Poverty, depression, and anxiety: causal evidence and mechanisms. Science.

[5] Kroenke K, Spitzer RL, Williams JB (2001). The PHQ-9: validity of a brief depression severity measure. J Gen Intern Med..

[6] Huang FY, Chung H, Kroenke K, Delucchi KL, Spitzer RL (2006). Using the Patient Health Questionnaire-9 to measure depression among racially and ethnically diverse primary care patients. J Gen Intern Med..

[7] Patel JS, Oh Y, Rand KL (2019). Measurement invariance of the Patient Health Questionnaire-9 (PHQ-9) depression screener in U.S. adults across sex, race/ethnicity, and education level: NHANES 2005–2016. Depress Anxiety..

[8] Levis B, Benedetti A, Thombs BD, for the DEPRESsion Screening Data (DEPRESSD) Collaboration (2019). Accuracy of Patient Health Questionnaire-9 (PHQ-9) for screening to detect major depression: individual participant data meta-analysis. BMJ.

[9] Levis B, Sun Y, He C (2020). Accuracy of the PHQ-2 alone and in combination with the PHQ-9 for screening to detect major depression: systematic review and meta-analysis. JAMA..

[10] Arges K, Assimes T, Bajaj V (2020). The Project Baseline Health Study: a step towards a broader mission to map human health. NPJ Digit Med..

[11] Armitage P (1955). Tests for linear trends in proportions and frequencies. Biometrics..

[12] Liu L, Berger VW, Hershberger SL. Trend tests for counts and proportions. Wiley StatsRef: Statistics Reference Online. 2014. 10.1002/9781118445112.stat06163.

[13] Mukaka MM (2012). A guide to appropriate use of correlation coefficient in medical research. Malawi Med J..

[14] Lockhart R, Taylor J, Tibshirani RJ, Tibshirani R (2014). A significance test for the LASSO. Ann Stat..

[15] Spitzer RL, Kroenke K, Williams JB (1999). Validation and utility of a self-report version of PRIME-MD: the PHQ primary care study. Primary Care Evaluation of Mental Disorders. Patient Health Questionnaire. JAMA..

[16] Siu AL, Bibbins-Domingo K, US Preventive Services Task Force (USPSTF) (2016). Screening for depression in adults: US Preventive Services Task Force Recommendation Statement. JAMA..

[17] de Vries McClintock HF, Bogner HR (2017). Incorporating patients’ social determinants of health into hypertension and depression care: a pilot randomized controlled trial. Community Ment Health J..

[18] Shittu RO, Issa BA, Olanrewaju GT, Mahmoud AO, Odeigah LO, Sule AG (2014). Social determinants of depression: social cohesion, negative life events, and depression among people living with HIV/AIDS in Nigeria, West Africa. Int J MCH AIDS..

[19] World Health Organization (WHO). Social Determinants of Mental Health. WHO web site. https://apps.who.int/iris/bitstream/handle/10665/112828/9789241506809_eng.pdf;jsessionid=16AAB9EC92EC506C097E56D0C8481D3F?sequence=1. Published 2014. Accessed April 22, 2021.

[20] Loprinzi PD, Davis RE (2016). Psycho-socioeconomic bio-behavioral associations on all-cause mortality: cohort study. Health Promot Perspect.

[21] Cao C, Hu L, Xu T (2020). Prevalence, correlates and misperception of depression symptoms in the United States, NHANES 2015–2018. J Affect Disord..

[22] Chobufo MD, Khan S, Agbor VN (2020). 10-Year trend in the prevalence and predictors of depression among patients with heart failure in the USA from 2007–2016. Int J Cardiol..

[23] Galenkamp H, van Oers H, Stronks K (2020). To what extent do socioeconomic inequalities in SRH reflect inequalities in burden of disease? The HELIUS study. J Public Health (Oxf)..

[24] Han K-M, Chang J, Yoon H-K (2019). Relationships between hand-grip strength, socioeconomic status, and depressive symptoms in community-dwelling older adults. J Affect Disord..

[25] Ettman CK, Cohen GH, Abdalla SM, Galea S (2020). Do assets explain the relation between race/ethnicity and probable depression in U.S. adults?. PLoS One.

[26] Assari S. Black Americans may be more resilient to stress than White Americans. Institute for Healthcare Policy & Innovation, University of Michigan web site. https://ihpi.umich.edu/news/black-americans-may-be-more-resilient-stress-white-americans. Published September 16, 2016. Accessed May 3, 2021.

[27] Fareed N, Swoboda CM, Jonnalagadda P, Huerta TR (2021). Persistent digital divide in health-related internet use among cancer survivors: findings from the Health Information National Trends Survey, 2003–2018. J Cancer Surviv..

