# Supplementary material for: Importance of Social Determinants in Screening for Depression
Source: J Gen Intern Med. 2021 Aug 17;37(11):2736–43. doi: 10.1007/s11606-021-06957-5 (PMC9411454; doi:10.1007/s11606-021-06957-5)
Supplement: Supplementary file 1 — (DOCX 27 kb) [file 11606_2021_6957_MOESM1_ESM.docx]

**Supplemental Material**

eTable 1.1 Demographic and Baseline Characteristics

eTable 1.2. On-Site Assessments: Physical Health Metrics

eTable 1.3. On-Site Assessments: Medical Conditions (60 most commonly reported), Symptoms (50 most commonly reported), and Allergies

eTable 1.4. On-Site Assessments: Mental Health Surveys

eTable 1.5. Blood Draw - Standard Laboratory Data

eTable 1.6. Participant Portal (App) Surveys

eTable 1.7. Sensors Data

eTable 2. PHQ-9 Social Determinants Linear Results

**eTable 1.1 Demographic and Baseline Characteristics**

| **Variable Name** | **Definition** |
| --- | --- |
| Age | Age in years at baseline |
| Sex | Self-reported sex at birth |
| Race | Self-reported race |
| Ethnicity | Self-reported Hispanic ancestry (yes/no) |
| Smoking Status | Self-reported smoking status at baseline |
| Pack-Years Smoked | Self-reported cigarettes smoked per day multiplied by years of regular smoking divided by 20 |

**eTable 1.2. On-Site Assessments: Physical Health Metrics**

| **Variable Name** | **Definition** |
| --- | --- |
| Systolic Blood Pressure | Average of 2 systolic blood pressure readings (mm Hg) measured at baseline |
| Diastolic Blood Pressure | Average of 2 diastolic blood pressure readings (mm Hg) measured at baseline |
| Heart Rate | Heart rate (beats/min) measured at baseline |
| Body Mass Index | Body weight (kg) divided by the square of height (cm) at baseline |
| Waist Circumference | Waist circumference (cm) at baseline |
| Oxygen Saturation | Oxygen saturation (%) at baseline |
| Respiratory Rate | Respiratory rate (breaths/min) at baseline |
| Ankle Brachial Index | Minimum of left and right ankle brachial index at baseline, using the maximum of left dorsalis pedis pressure and left posterior tibial pressure, maximum of right dorsalis pedis pressure and right posterior tibial pressure, and maximum of right and left brachial systolic pressure |
| 6-Minute Walk Distance | Total meters walked during 6-minute walk distance test at baseline |
| 10-Meter Walk Speed | Walking speed in meters per second using average of 3 fast walk trials during 10-meter walk test at baseline |
| Hand Grip | Average of 3 hand grip trials of dominant hand in kilograms at baseline |
| Single-Legged Balance | Average of left and right leg trials in seconds during single-legged balance test at baseline |
| Sitting-Rising Score | Sum of sitting and rising scores during sitting-rising test at baseline |
| 30-Second Chair Stand | Number of stands during 30-second chair stand test at baseline |
| Forced Expiratory Volume 1 (FEV1) / Forced Vital Capacity (FVC) Ratio | Ratio of forced expiratory volume (first third of forced breath) and forced vital capacity at baseline |
| Left Ventricular Ejection Fraction | Best available left ventricular ejection fraction from biplane, single, and visual read data from resting echocardiogram at baseline |
| Left Ventricular Mass Index | Left ventricular mass index from resting echocardiogram at baseline |
| Coronary Calcium Score | Coronary calcium score from coronary calcium scan at baseline |

**eTable 1.3. On-Site Assessments: Medical Conditions (60 most commonly reported), Symptoms (50 most commonly reported), and Allergies**

| **Variable Name** | **Definition** |
| --- | --- |
| Medical conditions |  |
| Osteoarthritis | Self-reported osteoarthritis at baseline |
| GERD | Self-reported gastroesophageal reflux disease at baseline |
| Hypertension | Self-reported hypertension at baseline |
| Asthma | Self-reported asthma at baseline |
| Cataracts | Self-reported cataracts at baseline |
| Hypercholesterolemia | Self-reported hypercholesterolemia at baseline |
| Migraines | Self-reported migraine headaches at baseline |
| Type II Diabetes | Self-reported type II diabetes at baseline |
| Sleep Apnea | Self-reported sleep apnea at baseline |
| Colon Polyps | Self-reported colon polyps at baseline |
| Pneumonia | Self-reported pneumonia |
| Hypothyroidism | Self-reported hypothyroidism at baseline |
| Severe Hearing Loss | Self-reported severe hearing loss at baseline |
| Kidney or Bladder Stones | Self-reported kidney or bladder stones at baseline |
| Arrhythmia | Self-reported arrhythmia at baseline |
| Gallbladder Disease | Self-reported gallbladder disease at baseline |
| Tinnitus | Self-reported tinnitus at baseline |
| Irritable Bowel Disorder | Self-reported irritable bowel disorder at baseline |
| Osteopenia | Self-reported osteopenia at baseline |
| Hyperlipidemia | Self-reported hyperlipidemia at baseline |
| Non-melanoma skin cancer | Self-reported non-melanoma skin cancer at baseline |
| Osteoporosis | Self-reported osteoporosis at baseline |
| Hemorrhoids | Self-reported hemorrhoids at baseline |
| Gout | Self-reported gout at baseline |
| Glaucoma | Self-reported glaucoma at baseline |
| Benign Prostatic Hyperplasia | Self-reported benign prostatic hyperplasia at baseline |
| Diverticulosis | Self-reported diverticulosis at baseline |
| Insomnia | Self-reported insomnia at baseline |
| Peptic Ulcers | Self-reported peptic ulcers at baseline |
| Melanoma Skin Cancer | Self-reported melanoma skin cancer at baseline |
| Diverticulitis | Self-reported diverticulitis at baseline |
| Myocardial Infarction | Self-reported myocardial infarction at baseline |
| Breast Cancer | Self-reported breast cancer at baseline |
| Chronic Obstructive Pulmonary Disease (COPD) | Self-reported COPD (with emphysema) at baseline |
| Psoriasis | Self-reported psoriasis at baseline |
| Coronary Artery Disease | Self-reported coronary artery disease (including angina) at baseline |
| Fibromyalgia | Self-reported fibromyalgia at baseline |
| Rheumatoid Arthritis | Self-reported rheumatoid arthritis at baseline |
| Pulmonary Embolism (PE) or Deep Vein Thrombosis (DVT) | Self-reported PE or DVT at baseline |
| Chronic Headaches | Self-reported chronic headaches (non-migraines) at baseline |
| Epilepsy | Self-reported epilepsy |
| Hashimoto’s Disease | Self-reported Hashimoto’s disease |
| Peripheral vascular disease | Self-reported peripheral vascular disease |
| Prostate cancer | Self-reported prostate cancer |
| Non-alcoholic fatty liver disease | Self-reported non-alcoholic fatty liver disease |
| Goiter | Self-reported goiter |
| Hepatitis C | Self-reported Hepatitis C |
| Diabetes type 1 | Self-reported Diabetes type 1 |
| Macular degeneration | Self-reported macular degeneration |
| Stroke | Self-reported stroke |
| Atrial fibrillation | Self-reported atrial fibrillation |
| Transient ischemic attack | Self-reported transient ischemic attack |
| Hepatitis B | Self-reported Hepatitis B |
| Symptoms | |
| Neck or Low Back Pain | Self-reported neck or low back pain at baseline |
| Stiffness | Self-reported stiffness at baseline |
| Muscle or Joint Pain | Self-reported muscle or joint pain at baseline |
| Nasal Stuffiness | Self-reported nasal stuffiness at baseline |
| Backache | Self-reported backache at baseline |
| Runny Nose | Self-reported runny nose at baseline |
| Urination at Night | Self-reported urination at night at baseline |
| Headache | Self-reported headache at baseline |
| Floaters | Self-reported floaters at baseline |
| Joint Pain Swelling | Self-reported joint pain swelling at baseline |
| Pain or Stiffness in the Neck | Self-reported pain or stiffness in neck at baseline |
| Itching | Self-reported itching at baseline |
| Cough | Self-reported cough at baseline |
| Dryness | Self-reported dryness at baseline |
| Easy Bruising or Bleeding | Self-reported easy bruising or bleeding at baseline |
| Tension | Self-reported tension at baseline |
| Tingling or Numbness in Extremities | Self-reported tingling or numbness in extremities at baseline |
| Tingling or Pins and Needles | Self-reported tingling or pins and needles at baseline |
| Bloating | Self-reported bloating at baseline |
| Heartburn | Self-reported heartburn at baseline |
| Frequency of Urination | Self-reported frequency of urination at baseline |
| Constipation | Self-reported constipation at baseline |
| Leg Cramps | Self-reported leg cramps at baseline |
| Diarrhea | Self-reported diarrhea at baseline |
| Ear Ringing | Self-reported ear ringing at baseline |
| Heat or Cold Intolerance | Self-reported heat or cold intolerance at baseline |
| Night Sweats | Self-reported night sweats at baseline |
| Dry Mouth | Self-reported dry mouth at baseline |
| Excessive Belching or Passing of Gas | Self-reported excessive belching or Passing of gas at baseline |
| Shortness of Breath with Exercise | Self-reported shortness of breath with exercise at baseline |
| Memory Change | Self-reported memory change at baseline |
| Lightheadedness | Self-reported lightheadedness at baseline |
| Sinus Pain | Self-reported sinus pain at baseline |
| Shortness of Breath | Self-reported shortness of breath at baseline |
| Swelling in Calves or Feet | Self-reported swelling in calves or feet at baseline |
| Coughing up Sputum | Self-reported coughing up sputum at baseline |
| Urgency | Self-reported urgency at baseline |
| Hay Fever | Self-reported hay fever at baseline |
| Discharge | Self-reported discharge at baseline |
| Body Image Concerns | Self-reported body image concerns at baseline |
| Hemorrhoids | Self-reported hemorrhoids at baseline |
| Cramping | Self-reported cramping at baseline |
| Numbness or Loss of Sensation | Self-reported numbness or loss of sensation at baseline |
| Wheezing | Self-reported wheezing at baseline |
| Allergies | |
| Any food allergies | Self-reported food allergies (any vs. none, ignoring additional details about which allergen) |
| Any seasonal allergies | Self-reported seasonal allergies (any vs. none, ignoring additional details about which allergen) |
| Any non-seasonal allergies | Self-reported non-seasonal allergies (any vs. none, ignoring additional details about which allergen) |
| Any medication allergies | Self-reported medication allergies (any vs. none, ignoring additional details about which allergen) |

**eTable 1.4. On-Site Assessments: Mental Health Surveys**

| **Variable Name** | **Definition** |
| --- | --- |
| Sheehan Disability Scale Score | Sheen Disability Scale total score (range 0, 30) at baseline |
| WHODAS 2.0 Score | WHODAS 2.0 total score (range 0, 48) at baseline |
| PHQ-9 Score | Patient Health Questionnaire-9 total score (range 0, 27) at baseline |
| GAD-7 Score | Generalized Anxiety Disorder-7 total score (range 0, 21) at baseline |
| BRFSS ACE Score | Behavioral Risk Factor Surveillance System Adverse Childhood Experience Module total problem count (range 0, 11) at baseline |

**eTable 1.5. Blood Draw - Standard Laboratory Data**

| **Variable Name** | **Definition** |
| --- | --- |
| Hemoglobin | Hemoglobin (g/dl) at baseline |
| Serum Creatinine | Serum Creatinine (mg/dl) at baseline |
| HDL | High density lipoprotein (mg/dl) at baseline |
| LDL | Low density lipoprotein (mg/dl) at baseline |
| Triglycerides | Triglycerides (mg/dl) at baseline |
| HbA1c | Hemoglobin A1c (%) at baseline |
| ALT | Alanine aminotransferase (U/L) at baseline |
| AST | Aspartate aminotransferase (U/L) at baseline |
| Vitamin D | Vitamin D (ng/ml) at baseline |
| CRP | C-reactive protein (mg/l) at baseline |
| Blood Glucose | Blood glucose (mg/dl) at baseline |
| Neutrophil / Lymphocyte Ratio | Ratio between neutrophils (k/mcL) and lymphocytes (k/mcL) at baseline |
| MCHC | Mean corpuscular hemoglobin concentration (% RBC) at baseline |
| Neutrophil Segments | Neutrophil segments (% WBC) at baseline |
| Total Neutrophils | Total neutrophils (% WBC) at baseline |
| Total Lymphocytes | Total lymphocytes (% WBC) at baseline |
| Magnesium | Magnesium (MEQ/L) at baseline |
| Monoocytes Percent | Monoocytes (% WBC) at baseline |
| Eosinophils Percent | Eosinophils (% WBC) at baseline |
| Basoophils Percent | Basoophils (% WBC) at baseline |
| Absolute Monocytes | Absolute Monocytes (k/mcL) at baseline |
| Absolute Eosinophils | Absolute Eosinophils (k/mcL) at baseline |
| Absolute Basophils | Absolute Basophils (k/mcL) at baseline |
| Hematocrit | Hematocrit (% RBC to whole blood volume) at baseline |
| MCV | Mean corpuscular volume (fL) at baseline |
| MCH | Mean corpuscular hemoglobin (pg) at baseline |
| MPV | Mean platelet volume (fL) at baseline |
| Platelet Count | Platelet count (cumm) at baseline |
| Albumin / Creatinine Ratio | Ratio between albumin and creatinine (mg/g) at baseline |
| RBC Count | Red blood cell count (millions/mcL) at baseline |
| WBC Count | White blood cell count (millions/mcL) at baseline |
| Calcium | Calcium (mg/dL) at baseline |
| Total Bilirubin | Total bilirubin (mg/dL) at baseline |
| Direct Bilirubin | Total direct bilirubin (mg/dL) at baseline |
| Cholesterol | Total cholesterol (mg/dL) at baseline |
| Lactic Dehydrogenase | Lactic dehydrogenase (U/L) at baseline |
| Globulin | Globulin (g/dL) at baseline |
| GGT | Gamma-glutamyl transferase (U/L) at baseline |
| Chloride | Chloride (MEQ/L) at baseline |
| Potassium | Potassium (MEQ/L) at baseline |
| Alkaline Phosphatase | Alkaline phosphatase (U/L) at baseline |
| Sodium | Sodium (MEQ/L) at baseline |
| Carbon Dioxide | Carbon dioxide (MEQ/L) at baseline |
| Inorganic Phosphorus | Inorganic phosphorus (mg/dL) at baseline |
| Protein (Serum) | Protein in serum (g/dL) at baseline |
| Albumin | Albumin (g/L) at baseline |
| Uric Acid | Uric acid (mg/dL) at baseline |
| Blood urea Nitrogen | Blood urea nitrogen (mg/dL) at baseline |
| Albumin (Urine) | Albumin in urine (mg/L) at baseline |
| Creatinine (Urine) | Creatinine in urine (mg/dL) at baseline |
| GFR MDRD | Glomerular filtration rate (mL/min/1.73 m^2^) based on Modification of Diet in Renal Disease Study equation at baseline |
| Free Thyroxine | Free thyroxine (ng/dL) at baseline |
| Absolute Reticulocytes | Absolute reticulocytes (billions/L) at baseline |
| Reticulocytes Percent | Reticulocytes (% RBC) at baseline |
| TSH | Thyroid stimulating hormone (mIU/L) at baseline |
| Urine Specific Gravity | Urine specific gravity at baseline |
| Urine Reaction pH | Urine reaction pH at baseline |

**eTable 1.6. Participant Portal (App) Surveys**

| **Variable Name** | **Definition** |
| --- | --- |
| PANAS Mood Positive Affect Score | PANAS Mood Positive Affect total score (range 10, 50) from first survey completed |
| PANAS Mood Negative Affect Score | PANAS Mood Positive Affect total score (range 10, 50) from first survey completed |
| Satisfaction with Life Score | Satisfaction with Life total score (range 5, 35) from first survey completed |
| Subjective Happiness Score | Subjective Happiness total score (range 4, 28) from first survey completed |
| AUDIT-C Score | Alcohol Use Disorders Identification Test-Concise total score (range 0, 12) from first survey completed |
| PROMIS Pain Intensity Score | PROMIS Pain Intensity total score (range 3, 15) from first survey completed |
| PROMIS Pain Interference Score | PROMIS Pain Interference total score (range 6, 30) from first survey completed |
| Perceived Social Support Score | Perceived Social Support total score (range 12, 84) at baseline from first survey completed |
| SES-Related Variables (Life Circumstances and Habits Survey) | |
| Highest Education Completed | Self-reported highest education level completed from first survey completed |
| Household Income | Self-reported household income from first survey completed |
| Marital Status | Self-reported marital status from first survey completed |
| Employment Status | Self-reported employment status from first survey completed |
| Health Insurance | Self-reported health insurance (yes/no) from first survey completed |

**eTable 1.7. Sensors Data**

| **Variable Name** | **Definition** |
| --- | --- |
| Mean daily steps first 30 days | Average daily number of steps in the first 30 days in study (measured with study watch) |
| Number of days watch worn 10+ hours in first 30 days | Number of days of 10 hours of wear in the first 30 days in study (measured with study watch) |

**eTable 2. PHQ-9 Social Determinants Linear Results**

| **Predictor** | **Beta** | **95% lower** | **95% upper** | **p-value** |
| --- | --- | --- | --- | --- |
| Age | -0.191426 | -0.278672 | -0.104181 | 0.000018 |
| Tension | 0.098218 | 0.029039 | 0.167396 | 0.005409 |
| Current smoker | 0.080003 | 0.012627 | 0.147378 | 0.019970 |
| Black race | -0.068353 | -0.133878 | -0.002827 | 0.040908 |
| Memory change | 0.065861 | -0.001389 | 0.133110 | 0.054921 |
| SES score | 0.065575 | 0.000302 | 0.130848 | 0.048950 |
| Body image concerns | 0.064142 | 0.000508 | 0.127777 | 0.048200 |
| Waist circumference | 0.062141 | -0.066843 | 0.191126 | 0.344891 |
| Former smoker | 0.048422 | -0.015687 | 0.112532 | 0.138706 |
| 6-minute walk | -0.041405 | -0.113206 | 0.030396 | 0.258253 |
| Backache | 0.041043 | -0.029714 | 0.111800 | 0.255461 |
| Kidney or bladder stones | -0.040898 | -0.101019 | 0.019224 | 0.182352 |
| Lightheadedness | 0.036874 | -0.026639 | 0.100387 | 0.255036 |
| Sleep apnea | 0.036549 | -0.026676 | 0.099775 | 0.257086 |
| Discharge | -0.034789 | -0.099142 | 0.029565 | 0.289221 |
| Diverticulitis | 0.032441 | -0.027189 | 0.092072 | 0.286155 |
| Handgrip | -0.031399 | -0.103570 | 0.040772 | 0.393672 |
| Mean daily steps | -0.030517 | -0.095875 | 0.034841 | 0.359969 |
| Asthma | 0.030425 | -0.030349 | 0.091198 | 0.326349 |
| Headache | 0.030143 | -0.035255 | 0.095541 | 0.366179 |
| Constipation | 0.029895 | -0.035251 | 0.095041 | 0.368283 |
| Respiratory rate | 0.028676 | -0.032869 | 0.090221 | 0.360984 |
| Migraines | 0.027246 | -0.035443 | 0.089935 | 0.394157 |
| Coughing up sputum | 0.026380 | -0.038316 | 0.091077 | 0.424029 |
| Food allergies | 0.026259 | -0.033645 | 0.086163 | 0.390113 |
| Coronary calcium score | -0.026184 | -0.091125 | 0.038757 | 0.429228 |
| Ejection fraction at rest | 0.025537 | -0.035014 | 0.086087 | 0.408315 |
| Shortness of breath with exercise | 0.025479 | -0.049744 | 0.100702 | 0.506629 |
| Joint pain swelling | 0.024825 | -0.039596 | 0.089246 | 0.449923 |
| Neck or low back pain | 0.024820 | -0.044730 | 0.094371 | 0.484126 |
| Gallbladder disease | -0.024484 | -0.086240 | 0.037272 | 0.436968 |
| Nonmelanoma skin cancer | 0.023442 | -0.037718 | 0.084602 | 0.452362 |
| Nasal stuffiness | 0.023163 | -0.042330 | 0.088655 | 0.488046 |
| Irritable bowel disorder | 0.023110 | -0.037509 | 0.083730 | 0.454783 |
| Cataracts | 0.022960 | -0.048319 | 0.094240 | 0.527674 |
| Hypothyroidism | -0.022671 | -0.083638 | 0.038296 | 0.465960 |
| Osteoarthritis | 0.020420 | -0.048836 | 0.089676 | 0.563201 |
| Heat or cold intolerance | 0.019377 | -0.045998 | 0.084751 | 0.561156 |
| GERD | 0.018891 | -0.044179 | 0.081960 | 0.557024 |
| 30 sec chair stand | -0.018589 | -0.087304 | 0.050126 | 0.595831 |
| Heart rate | 0.016639 | -0.047475 | 0.080752 | 0.610871 |
| Night sweats | 0.016587 | -0.045934 | 0.079108 | 0.602944 |
| BMI | 0.014935 | -0.109289 | 0.139159 | 0.813641 |
| Asian race | -0.012736 | -0.074835 | 0.049363 | 0.687600 |
| Pneumonia | 0.011978 | -0.049446 | 0.073402 | 0.702211 |
| Epilepsy | 0.011854 | -0.047623 | 0.071332 | 0.695955 |
| COPD w/ emphysema | 0.010224 | -0.057619 | 0.078066 | 0.767629 |
| Shortness of breath | 0.009791 | -0.068785 | 0.088367 | 0.806988 |
| AUDIT-C | 0.009699 | -0.052507 | 0.071906 | 0.759819 |
| Bloating | -0.007558 | -0.074109 | 0.058993 | 0.823789 |
| Fibromyalgia | 0.007187 | -0.054050 | 0.068423 | 0.818004 |
| Pulmonary embolism | 0.006214 | -0.053389 | 0.065818 | 0.838021 |
